# Viral load change and time to death among adult HIV/AIDS patients on ART after test-and-treat in Northwest Ethiopia: a retrospective multi-center follow-up study using Bayesian joint modeling

**DOI:** 10.3389/fpubh.2025.1418999

**Published:** 2025-03-18

**Authors:** Eyob Tilahun Abeje, Eskezyiaw Agedew, Bekalu Endalew, Gedefaw Diress Alen

**Affiliations:** ^1^Department of Epidemiology and Biostatistics, School of Public Health, College of Medicine and Health Sciences, Wollo University, Dessie, Ethiopia; ^2^Department of Public Health, College of Health Sciences, Debre Markos University, Debre Markos, Ethiopia

**Keywords:** viral load change, viral load pattern, time to death, HIV/AIDS, viral load rebound, survival analysis, Bayesian analysis

## Abstract

**Introduction:**

Among patients infected with Human Immunodeficiency Virus who are on antiretroviral therapy, nearly one-fifth develop viral load rebound within 2 years of initiation of therapy. Studies on viral load change are limited in Ethiopia. Previous studies have not adequately accounted the undetectable viral load in the analysis and the association between viral load change and time to death. This study assessed viral load change, its predictor variables, and the joint association between viral load change and time to death.

**Methods:**

An institution-based retrospective follow-up study was conducted. The data were extracted from 24 April to 30 May 2022 using charts of 489 study participants selected using simple random sampling. OpenBUGS software from the R2OpenBUGS R package was used for model building. A joint Tobit skewed normal mixed effects model and survival analysis using a Bayesian approach was employed.

**Results:**

The data were extracted from a total of 489 participants. Starting from six months post-treatment initiation (time zero), the log viral load decreased by 0.027 log units per month until 10.82 months of follow-up, while after 20.9 months, it increased by 0.034 log units per month. Participants who took ART medication outside of the catchment health facility had 0.29 log viral load unit higher than within the catchment health facility. The hazard of death was 3.5 times higher for individuals whose log viral load slope increased by one standard deviation from the population slope during the first 10.82 months of follow-up.

**Conclusion:**

The change in log viral load increment was high during the latter follow-up period compared to the decrement in log viral load at the beginning of the follow-up period. Duration of treatment, taking ART medication outside the catchment area, baseline WHO stage three and four, poor adherence were associated with log viral load change. Addressing stigma and discrimination is essential to prevent ART patients from seeking treatment outside the catchment area, improve treatment outcomes and reduce viral load rebound.

## Introduction

Human Immunodeficiency Virus/Acquired Immunodeficiency Syndrome (HIV/AIDS) has become a global pandemic, infecting and killing millions since the 1980s. It destroys the immune system progressively and results in a condition called AIDS (1, 2). Highly Active Antiretroviral Therapy (HAART) has been taken by an estimated 27.5 million people living with HIV globally by the end of 2020. However, HIV remains a global health crisis: the world saw 1.5 million new HIV infections and 680,000 deaths from AIDS-related causes that occurred in 2020 (3, 4). In Ethiopia, besides the HAART, Pre-exposure prophylaxis (PrEP) for HIV is used by individuals who are not infected with HIV but are at a substantial risk to block the acquisition of HIV. HIV-negative female sexual workers (FSWs) and HIV-negative spouses of Sero-discordant couples are the intended recipients of PrEP services (5).

HIV viral load quantifies the amount of virus in the blood (viral RNA count in 1 mL of blood), which is then used to monitor treatment effectiveness and the progression of disease (1, 2). According to the 2021 World Health Organization (WHO) guidelines, routine viral load monitoring starts at 6 months after antiretroviral therapy (ART) initiation and is then followed up at 12 months and yearly thereafter for patients that have undetectable viral load until the occurrence of treatment failure. The new addition to this guidelines is the provision of enhanced adherence counseling if the viral load is between 50 and 1,000 copies/milliliter (ml) and repeat viral load testing after 3 months unlike the previous guidelines where enhanced adherence support has been done when viral load is greater than 1000 copies/ml (6, 7).

Now the world is aiming to achieve the 95–95-95 target for HIV issued by the Joint United Nations Program on HIV/AIDS (UNAIDS) on HIV/AIDS by the end of 2030. However, Ethiopia is one of the sub-Saharan countries short of achieving the 90–90-90 target with one-fourth of the population having a high viral load count (unsuppressed viral load) (3, 8). The median survival time was 8 years according to a meta-analysis study and only one-fourth survive past 12 years (9). In Ethiopia, the median survival time for HIV patients was three years, but this has improved in recent years. More than half of the participants were alive at the end of the follow-up period, with nearly equal follow-up durations observed due to the provision of free and accessible highly active antiretroviral therapy. Incidence of death was lowered from ten to three per hundred person years of follow-up for nearly equal length of follow-up time (10–14). However, nearly one out of five people die within 5 years of starting therapy even after the initiation of the test and treat program (13, 14). HIV/AIDS-related mortality has shown a declining trend over the years in Ethiopia. The decline was remarkable among the under-5 age group followed by the 15–49 age groups, whereas the age group 50–69 has shown an upward trend in recent years. In addition to this, the incidence-to-mortality ratio is now less than one due to high mortality (15).

To overcome this problem, there exist many interventions and strategies, from provision of higher-line therapies to the test and treat program in Ethiopia. Routine viral load testing and monitoring are done to identify treatment failure as early as possible and switch to higher-line therapy (6, 16–22). However, still nearly one-fifth of all patients in Ethiopia develop viral load rebound within 2 years of initiation of therapy, which in turn leads to treatment failure (drug resistance mutation) and death (23).

Different viral load measuring intervals based on the length of undetectable viral load and patient characteristics have shown better patient prognoses. However, in Ethiopia, emphasis and frequent measurements are given after they already have a high viral load count due to the cost of scaling up the viral load measurements (1, 6, 7, 18–21). After the provision of the test and treat program, viral load is used for treatment monitoring and treatment failure. However, there are study gaps on viral load change over time, predictors of viral load change, and the association between viral load change and time to death. Previous studies showed limitations, including an inability to incorporate undetectable viral load measurements into the analysis and had short follow-up time. This study has aimed to assess viral load change and its predictor variables as well as the joint association between viral load change and time to death among adult HIV/AIDS patients on ART after initiation of the test and treat program.

## Methods

### Study area and period

The study was conducted at three comprehensive specialized hospitals in the Amara region, Debre Markos, Debre Tabor, and Felege Hiwet comprehensive specialized hospitals in northwest Ethiopia. The patients newly registered for ART between 2017 and 2018 made up a total of 870 during the recruitment period. The study was conducted from 1 March 2017 to 13 April 2022. The data extraction period was from 24 April to 30 May 2022.

### Study design

An institution-based retrospective follow-up study was conducted.

### Population

#### Source population

All adult HIV/AIDS patients who newly started ART at Debre Markos, Debre Tabor, and Felege Hiwet comprehensive specialized hospitals after the initiation of the Universal Test and Treat (UTT) program were the source population.

#### Study population

All adult HIV/AIDS patients who newly started ART at Debre Markos, Debre Tabor, and Felege Hiwet comprehensive specialized hospitals after initiation of the UTT program from 1 March 2017 to 1 April 2018 were the study population.

### Eligibility criteria

#### Inclusion criteria

All adult HIV/AIDS patients who newly started ART at Debre Markos, Debre Tabor, and Felege Hiwet comprehensive specialized hospitals after initiation of UTT program and who had at least two total viral load measurements (the first viral load test at 6 months of treatment initiation and one additional viral load measurement) were included.

#### Exclusion criteria

Transferred-in patients after started treatment between March 01/2017 and April 01/2018 were excluded.

### Sample size determination and sampling technique

#### Sample size determination

The two-step sample size calculation approach method was used for this study (24).

#### Sample size calculation for viral load change

GLIMMPSE version 3.0.0 software was used to calculate the sample size for viral load change. Longitudinal mixed-effects regression model sample size calculation requires the effect size between the comparison groups (−0.1298), treatment regimen (AZT-3TC-NVP vs. TDF + 3TC + NVP), correlation structure (6 × 6 correlation matrix), number of measurements of the outcome variable (6), group ratio (1.5:1, AZT-3TC-NVP to TDF + 3TC + NVP), standard deviation ratio over time (1.0, 1.15, 1.0, 0.84, 0.7, 0.8), within individuals variance of the outcome variable (0.4115), power (80%), and confidence level (95%) (25, 26). These parameters were obtained from a study conducted at Zewuditu Hospital (27). The largest sample size was obtained from the effect size of the treatment regimen (AZT-3TC-NVP vs. TDF + 3TC + NVP), and the sample size was 143. The treatment regimen variable has four categories: 25% of the sample was added and it became 179 (26). $N=\frac{n_{0}}{\left(1-q\right)}$, where $n_{0}$ is the sample size through the assumption of complete cases = 179; q is the missing data due to death and loss to follow up from a study conducted at Addis Ababa after universal test and treat (=0.21 death +0.164 lost to follow-up = 0.374).$N=\frac{179}{\left(1-0.37\right)}$=286, and after the addition of 15% for incomplete data, it became 329.

#### Sample size calculation for survival data

For time-to-event data sample size calculation, the required parameters were significant effect size hazard ratio, event probability, withdraw probability, survival probability, group size ratio, confidence level, and power (28, 29). This information was obtained from a previous study in Addis Ababa on time to death after a universal test and treat program in public health hospitals (14). Stata 14 was used for sample size calculation. The event probability and withdrawal probability conducted in Addis Ababa after initiation of the test and treat program were 21.1 and 16.4%, respectively (14). The largest calculated sample size was obtained from the baseline CD4 independent variable (baseline CD4 200–350cells/mm3 versus baseline CD4 less than 200cells/mm3). Then, power logrank 0.84, hratio(0.257) power(0.8) wdprob(0.164) nratio(0.48) became 366. Because baseline CD4 was categorized into four groups, the sample size was increased by 25%, resulting in a final sample size of 458 (26). With 15% data incompleteness, it became 527. Then, the calculated sample size in the second stage was higher than the first stage (24). Therefore, the final sample size was declared to be 527.

### Sampling technique and procedure

A simple random sampling technique was used. First, the number of newly registered patients from 1 March 2017 to 13 April 2018 in the logbook who had viral load measurements taken at 6 month of treatment initiation was selected based on their Medical Record Number (MRN). A sampling frame was developed for identified cards and a random computer-generated number was used to select the sampling units. The sampling frame was prepared from the eligible participants of Felege Hiwot (387), Debre Markos (198), and Debre Tabor (152). The final sample comprised 268 samples from Felege Hiwet, 145 from Debre Markos and 114 from Debre Tabor comprehensive specialized hospital.

### Study variables

#### Outcome variables

The outcome variables were viral load change and time to death.

#### Independent variables

These are independent variables for both time to death and viral load change:

Demographic characteristics - gender, age, religion, marital status, employment, educational status.

Clinical characteristics - Cluster of Differentiation 4 (CD4), WHO clinical stage, baseline CD4, adherence, Co-trimoxazole Preventive Therapy (CPT), opportunistic infection, Tuberculosis (TB), anemia, Isoniazid Preventive Therapy (IPT), Body Mass Index (BMI). and weight.

#### Operational definition

##### Adherence

The extent to which a person’s behavior on taking medications, following a diet, and executing lifestyle changes corresponds with agreed recommendations from a health care provider (7, 30):

Good- taken greater than 95% doses of prescribed drugs,

Fair- taken 85–94% doses of prescribed drugs, and.

Poor- taken less than 85% doses of prescribed drugs.

##### Catchment area

The geographic region served by a specific health facility, where patients receive ART (7, 12).

*Within Catchment Area*: Patients who receive ART at the nearest health facility designated for their region.

*Outside Catchment Area*: Patients who receive ART at a health facility other than the one nearest to them.

##### Treatment initiation

It refers to the time point at which participant commences their antiretroviral therapy (ART) regimen. This time point serves as the baseline for variables specifically measured at the start of treatment (5).

##### Six months post-treatment initiation

It refers to the time point at six months after the commencement of ART. At this time point, the initial viral load measurement was obtained to assess subsequent changes in viral load and treatment outcomes over time. Time zero for this study was set at six months post-treatment initiation. The follow-up time was measured starting from this point (5, 59).

### Data extraction tools and procedure

The data extraction tool was developed from different kind of literature (10, 12, 27, 31–33). Three components made up the data extraction: clinical, behavioral, and demographic characteristics. Data extractors looked over the patient’s card and the information was recorded into the data extraction tool until the patient passed away, was lost, transferred out, or the follow-up time ended.

### Data quality management

The data extraction tool was tested for the availability of identified variables 2 weeks before the main data extraction period from 10 MRN charts at Debre Markos compressive specialized hospital on 18 April 2022. Based on the information gathered from the pretest, the data extraction tool was modified for main data extraction. The day before data extraction at each center, supervisors and data extractors attended a one-day training session. Supervisors and investigators reviewed and coded the completed data daily.

### Data processing and analysis

The data was checked, refined, and entered into Epi-Data version 3.1 and imported to R software version 4.1.2 for graphical analysis and using OpenBUGS software from R2OpenBUGS R package for model building. Descriptive statistical analysis was done first, followed by Tobit skewed normal mixed effects model and piecewise constant cox proportional hazard model were analyzed separately. Finally, joint Tobit skewed normal mixed effects model and survival analysis was performed. The Bayesian Markov Chain Monte Carlo (MCMC) approach was used for estimation (34–40). DIC (deviance information criteria) was used for model selection. Finally, model diagnostics was done using posterior predictive model checking (29, 41–51). Improper (vague) priors were used for all parameters. The DIC was set after checking the convergence assessment based on Gelman and Rubin diagnostic plot, trace, history, and autocorrelation plots. A total of 125,000 initial iterations were used until all the parameters mixed well. This iteration was then discarded as burn-in and an additional 100,000 iterations were run before being declared as the final output. The Monte Carlo standard errors were less than 1/50th of the standard deviation for all the parameters.

### Ethical approval and waived informed consent

Ethical approval was obtained from the Institutional Review Committee of the College of Health Sciences, Debre Markos University (Ref.No/HSC/R/C /Ser/co/101/ 11/14). Waived informed consent was obtained from the Institutional Review Committee of the College of Health Sciences, Debre Markos University, as it involved secondary data from the patients’ charts. By collecting data in an entirely anonymous manner and not including the patients’ names or MRN on the data extraction checklist, confidentiality of the information was guaranteed. Ultimately, the dataset was merely used for this study analysis. Every technique and material used was carried out in compliance with the instructions.

## Results

The data was extracted from 489 participants with a data completeness rate of 92.7%.

### Socio-demographic characteristics

Of the study participants, 53.2% were female, 48.3% were married, and 17.0% of the participants came from outside the catchment area. The mean age of the participants was 37.67 years with a standard deviation of 11.08 (Table 1).

**Table 1:** Socio-demographic characteristics.

Categorical variables		Proportion
Sex	Male	229 (46.83%)
	Female	260 (53.17%)
Occupation	Daily labor	89 (18.2%)
	Farmer	34 (7.0%)
	Government employee	91 (18.6%)
	Housewife	43 (8.8%)
	Merchant	103 (21.1%)
	Private	97 (19.8)
	Other	32 (6.5%)
Marital status	Never married	126 (25.8%)
	Married	214 (43.8%)
	Divorced	109 (22.3%)
	Widowed	40 (8.1%)
Religion	Orthodox	447 (91.4%)
	Protestant	11(2.3%)
	Muslim	31(6.3%)
Education status	No education	89 (18.2%)
	Prime education	130 (26.6%)
	Secondary education	142 (29.0%)
	Higher level	128 (26.2)
Catchment area	Outside	83 (17.0%)
	Within	406 (83.0%)

Continuous variable		
	Mean	Standard deviation
Age	37.67	11.08

### Clinical characteristics

Of the study participants, 6.3% had poor adherence and 16.8% had fair. The mean weight of the participants was 52.44 with a standard deviation of 7.41 (Table 2).

**Table 2:** Clinical characteristics.

Clinical characteristics		Proportion
Baseline WHO	Stage 1	145 (29.7%)
	Stage 2	179 (36.6%)
	Stage 3	142 (29.0%)
	Stage 4	23 (4.7%)
CPT	No	260 (53.2%) 229
	Yes	229 (46.8%)
Baseline tuberculosis	No	458 (93.7%)
	Yes	31 (6.3%)
IPT	No	31 (6.3%)
	Yes	458 (93.7%)
Baseline regimen	AZT-3TC-EFV	11 (2.2%)
	AZT-3TC-NVP	37 (7.6%)
	TDF-3TC-EFV	438 (89.6%)
	TDF-3TC-NVP	3 (0.6%)
Adherence	Good	376 (76.9%)
	Fair	82 (16.8%)
	Poor	31 (6.3%)

Clinical characteristics		
	Mean	Standard deviation
Baseline CD4	397.1	205.5
Baseline hemoglobin	13.4	1.59
Weight	52.44	7.41
Height	165.0	6.10
BMI	19.30	2.09

#### Outcome variables

Of the study participants, 6.95% died before the follow-up period ended, 18.0% were lost to follow up, and 4.1% were transferred out (Table 3).

**Table 3:** Outcome variables.

Variables		Proportion
Event status	Censor	455 (93.04%)
	Event	34 (6.95%)
Censor type	Lost	88 (18.0%)
	Period ends	355 (75.6%)
	Transfer out	20 (4.1%)

#### Survival probability

**Figure 1:**
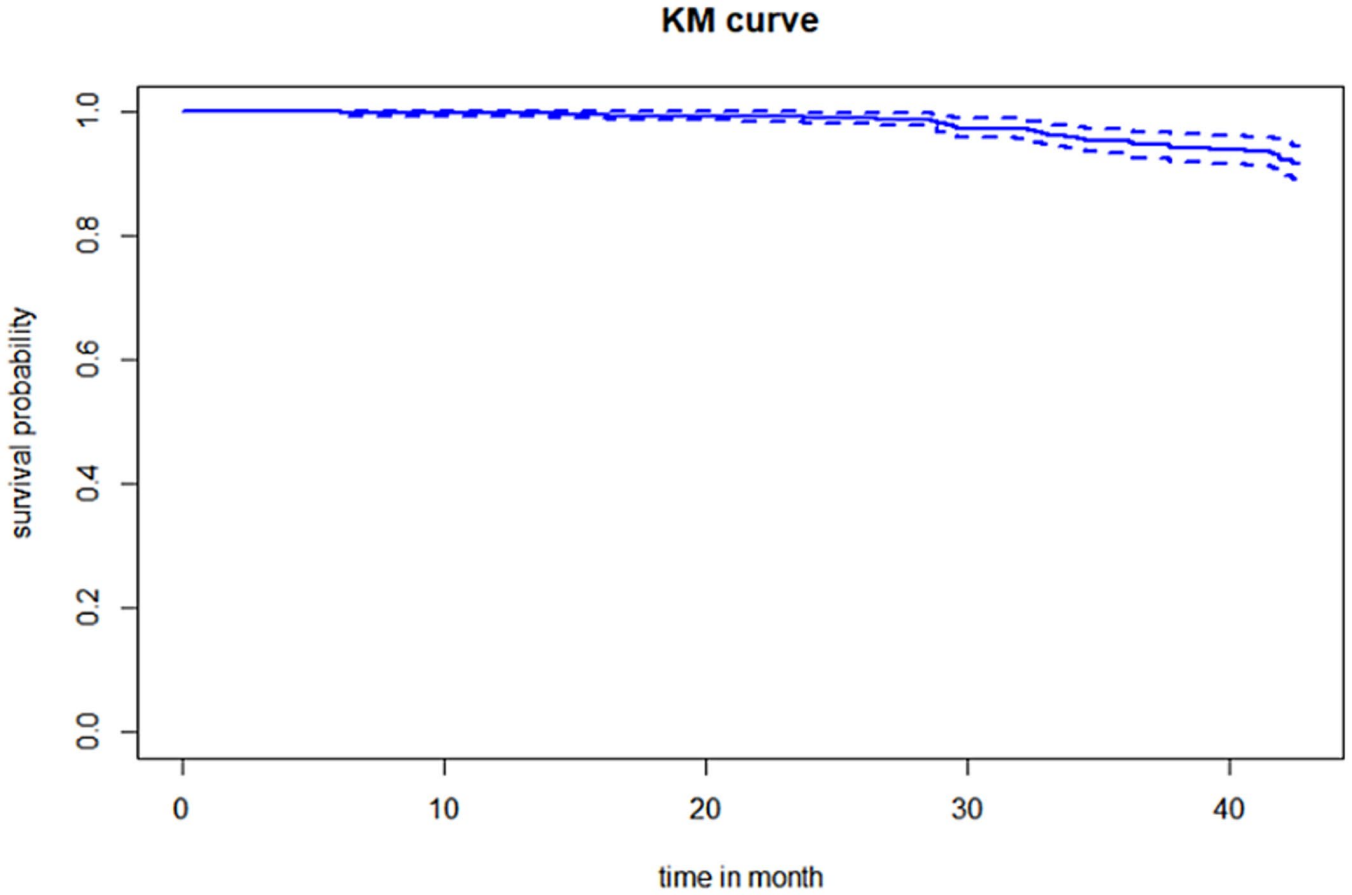
Survival probability.

The overall survival probability was 0.918 with a maximum of 42.667 months of follow-up. The restricted mean survival time was 41.9 (41.58, 42.2). Half of the deaths occurred after 25 months of follow-up (Figure 1).

### Explanatory analysis

#### Exploring viral load individual profile

**Figure 2:**
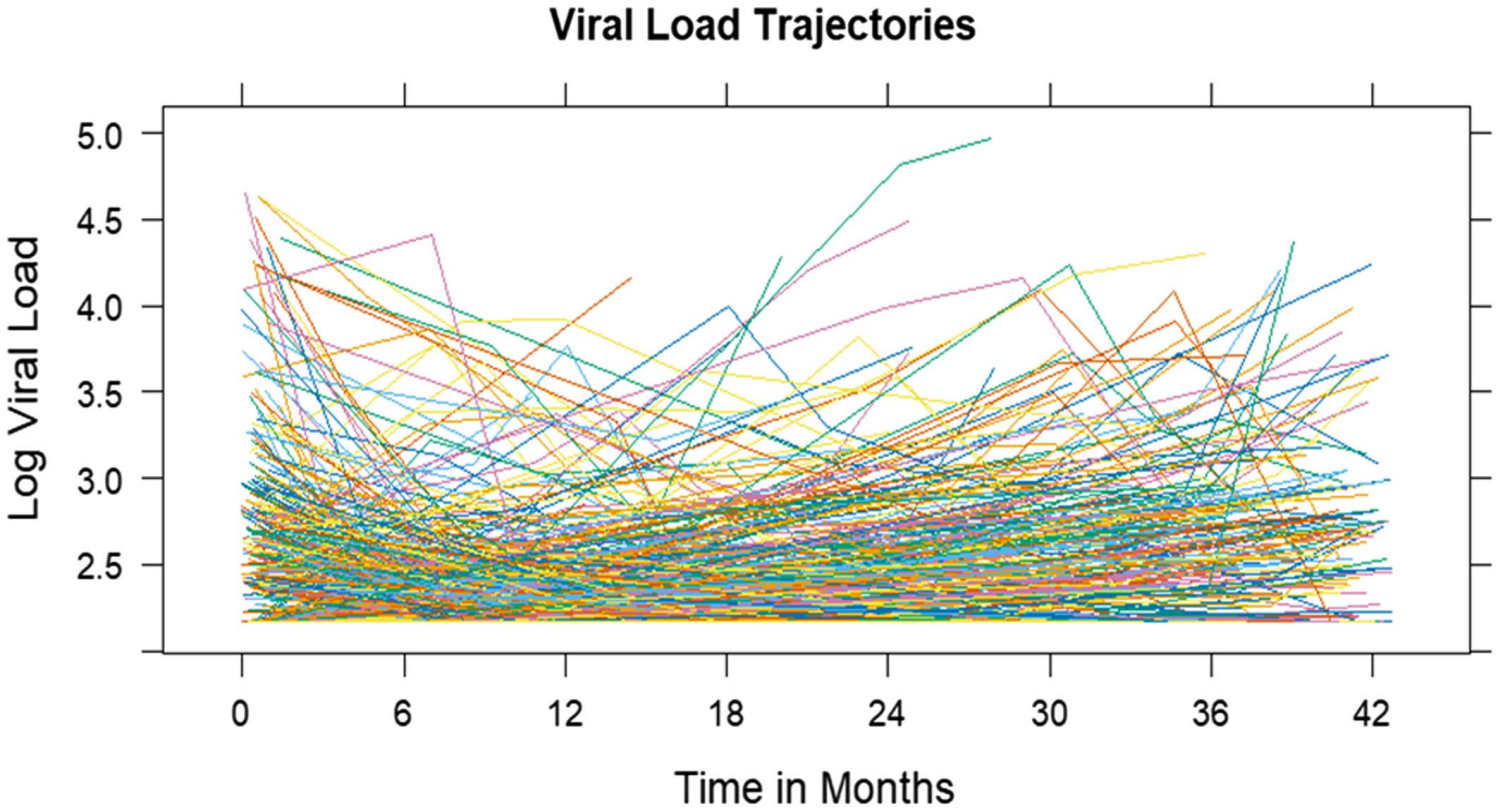
Viral load trajectory: individual profile plots.

Individual study participants had different viral loads at six months post-treatment initiation ranging from undetectable viral load to high viral load counts greater than 1,000 copies (random intercept). Individual viral load profiles show that individuals with high log viral load counts at 6 months of treatment saw a faster decrease in log viral load than low log viral load at 6 months of treatment (random slope) (Figure 2).

#### Exploring the mean

**Figure 3:**
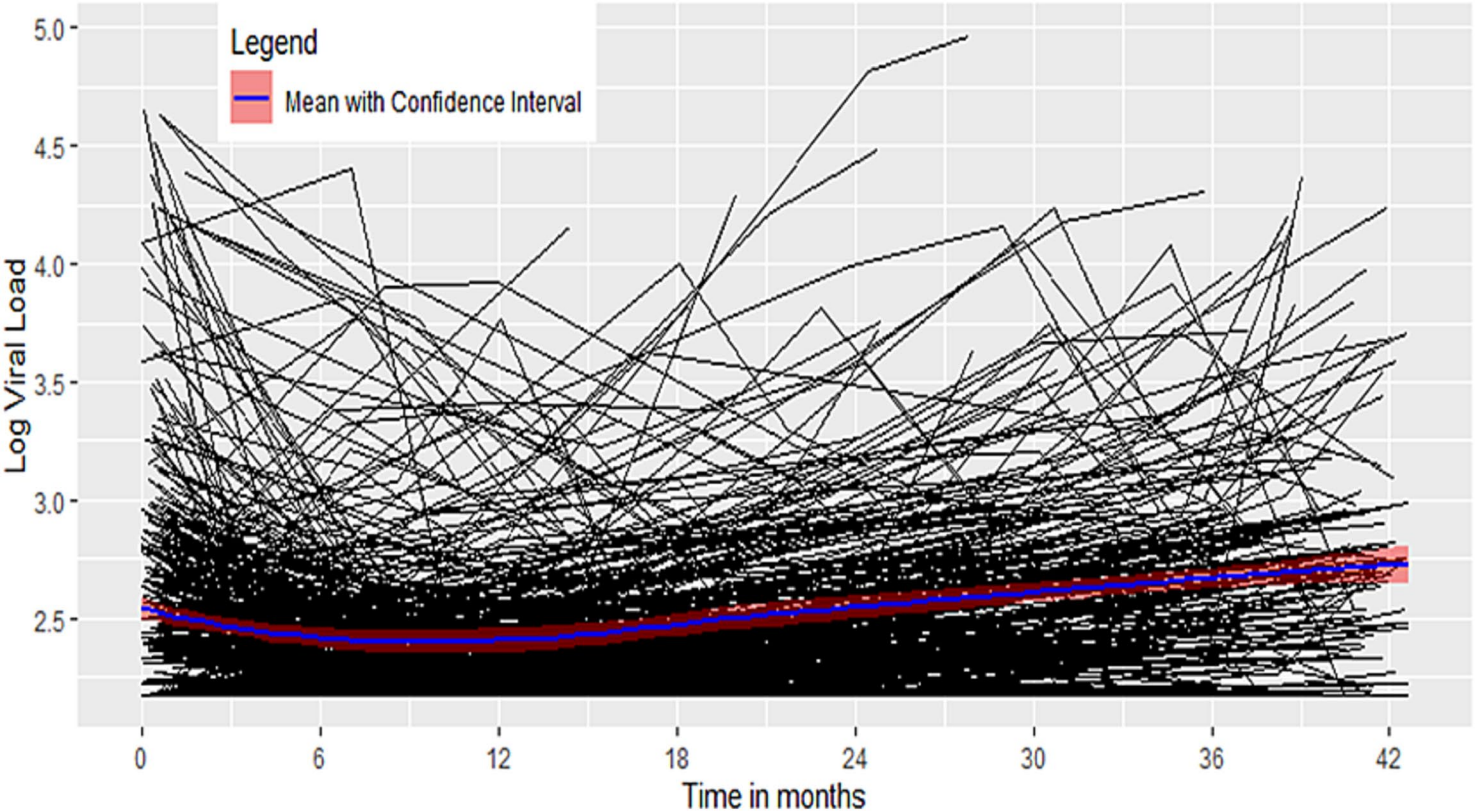
Mean structure using smoother.

The mean profile shows a decreasing trend at the beginning of the follow-up period, remaining low with a curved transition, and then an increasing trend during the latter follow-up period (52–54) (Figure 3).

#### Summarizing the data exploration

Different viral loads at the start of the study and different viral load progressions indicate a random intercept and random slope. The mean profile shows a linear spline with bent cable transition.

#### Viral load

From 1,485 measurements, 586 (40.2%) showed an undetectable viral load, 189(12.7%) showed a viral load greater than 1,000 copies, and the rest 700 (47.1%) showed viral load measurements that fell somewhere in between. The lower detection limit was 150 copies per ml. After applying log10 transformation to the viral load, the distribution exhibit half-right skewed normal distribution.

#### Final joint model

From all the variables in the descriptive analysis, baseline CD4, age of the participant, baseline WHO stage, catchment area, and adherence were included in the final joint model. The posterior mean 95% credible interval for the log viral load over time, both before and after the transition, indicates an association between time and log viral load. Log viral load decreased by 0.02675 (10^0.02675^ = 1.06 viral load copies) when time increased by 1 month up to 10.82 months. Log viral load increased by 0.03384 (10^0.03384^ = 1.08 viral load copies) when time increased by 1 month ((−0.02675) + 0.06059 = 0.03384) after 20.925 months of follow-up.

Participants who took ART medication outside of the catchment health facility had 0.29 log viral load unit higher than within the catchment health facility. Patients with WHO stages 3 and 4 showed an increase in the log viral load by 0.6078 and 0.738 respectively than WHO stage 1 at the start of the study. Poor adherence increases the hazard of death by 88.5% ($e^{0.634}=1.885\left(1.20,2.8\right)$) compared to good adherence. The hazard of death is increased by 1.34 times when the age of the patient increases by one standard deviation (11.08 years).

The hazard of death was 3.5 times higher for individuals whose log viral load slope increased by one standard deviation from the population slope during the first 10.82 months of follow-up (Table 4).

**Table 4:** Viral load change sub-model and time-to-death sub-model.

Longitudinal sub-model		Mean	Sd	val2.5pc	Median	val97.5pc
Variables						
Intercept		1.557	0.1351	1.285	1.559	1.817
Time (slope one)		−0.02675	0.01091	−0.04883	−0.02664	−0.005678
Time (slope two)		0.06059	0.01556	0.02935	0.06034	0.09157
Variance intercept		0.1018	0.03429	0.05138	0.09629	0.1879
Cov. intercept and slope one		−0.0091	0.003678	−0.01632	−0.008903	−0.00192
Cov. intercept and slope two		0.0068	0.007598	−0.006808	0.006328	0.0232
Variance slope one		0.0130	0.002132	0.009558	0.01278	0.01796
Cov. slope one and slope two		−0.0106	0.002692	−0.01687	−0.01031	−0.006301
Variance slope two		0.0215	0.003996	0.01522	0.02105	0.03084
Skewness		0.0034	0.148	−0.2849	0.002816	0.3013
Base CD4		−0.0678	0.07832	−0.2221	−0.06724	0.08427
Age		0.1297	0.07449	−0.01762	0.13	0.2734
Catchment area	Within	Ref				
	Outside	0.2853	0.1527	0.02398	0.2768	0.6043
Base WHO	Stage 1	Ref.				
	Stage 2	0.2154	0.1559	−0.08851	0.2161	0.5252
	Stage 3	0.6078	0.1876	0.2452	0.6075	0.9767
	Stage 4	0.738	0.2564	0.2304	0.7374	1.244
Adherence	Good	Ref.				
	Fair	0.2859	0.1641	−0.03908	0.2855	0.6092
	Poor	0.7316	0.2636	0.212	0.7297	1.25
Variance		1.23	0.08177	1.082	1.226	1.401
Transition parameters						
Gam		5.425	1.75	1.995	4.801	8.85
Kappa		1.864	0.3746	1.129	1.793	1.92
Tau		15.5	0.7335	14.06	15.71	16.93

Survival sub-model							
Variables		Mean	Sd	val2.5pc	Median	val97.5pc	Hazard ratio
Catchment area	Within	Ref.					
	Outside	−0.1252	0.1353	−0.3954	−0.123	0.1371	0.88(0.67–1.15)
Base WHO	Stage 1						
	Stage 2	0.276	0.205	−0.1142	0.2695	0.6927	1.32(0.89–1.99)
	Stage 3	0.664	0.2216	0.2414	0.6573	1.098	1.94(1.27–2.99)
	Stage 4	1.214	0.2018	0.8187	1.209	1.624	3.37(2.27–5.07)
Adherence	Good	Ref.					
	Fair	0.06223	0.1512	−0.2323	0.06025	0.3677	1.06(0.79–1.44)
	Poor	0.6341	0.2101	0.1898	0.6421	1.031	1.89(1.21–2.80)
Base CD4		−0.1043	0.08636	−0.2749	−0.1061	0.06679	0.90(0.76–1.07)
Age		0.291	0.06659	0.1561	0.2912	0.4215	1.34(1.17–1.52)
Association intercept		0.1061	0.5812	−1.0330	0.111	1.248	1.11(0.36–3.48)
Association slope one		1.249	0.506	0.257	1.181	2.241	3.5 (1.29–9.40)
Association slope two		0.650	1.132	−1.562	0.6445	2.88	1.92(0.21–17.63)

## Discussion

The log viral load showed a decreasing trend during the first eleven months of follow-up. This is supported by studies conducted in the United States, sub-Saharan Africa, Cameron, Arbanch, and Zewuditu Memorial Hospital in Ethiopia (27, 32, 55–59). This may be due to the HAART and adherence support. The log viral load remains low during the middle of the follow-up period. This is supported by studies done in the United States and sub-Saharan Africa (55, 56). This may be because better adherence to treatment and the effectiveness of HAART in reducing viral loads to their lowest achievable levels, beyond which further reduction is not possible under the current treatment regimen and guidelines.

The log viral load increases after 21 months of initiation of the study. This finding is supported by studies done in the United States and sub-Saharan Africa (55, 56). This may be due to treatment failure and non-adherence at the latter follow-up time. There is a discrepancy between this study and studies done in Arbanch and Zewuditu, Cameron, South Africa Memorial Hospital in Ethiopia (27, 32, 57–59). They concluded the viral load was decreasing over time. This may be due to methodological differences in the longitudinal growth curve model, inclusion of skewness, assumption of missing data, and incorporating undetectable viral load measures in this study. Even though the viral load follows similar trend, the effect size in this study is lower than the previous studies with the same pattern, mainly before the transition parameter. This may be because this study’s viral load measurements were taken 6 months after treatment initiation. The viral load is low due to HAART and more frequent viral load monitoring in this study than in previous studies.

The correlation between baseline log viral load and log viral load change before 10.82 months of follow-up is negative. This finding is supported by studies done in South Africa and Zewuditu Memorial Hospital (27, 32, 59). This may be because individuals with a high viral load experience a more rapid decrease over time due to treatment effects and adherence to medication compared to those with a low viral load at six months post-treatment initiation. The correlation between log viral load change before 10.82 months of follow-up and after 20.92 months of follow-up is strongly negative. This may be because having a high viral load before transition becomes having a low viral load after a while due to adherence counseling and a next-line treatment switch.

Higher WHO stages (3 and 4) are associated with higher log viral load at six months post-treatment initiation. This finding is supported by studies done in Ethiopia at Zewuditu Memorial Referral Hospital, South Africa, sub-Saharan Africa (27, 32, 56). This may be due to the log viral load remaining high for those with advanced WHO stages even 6 months after initiation of treatment. On the other hand, there is a discrepancy between this study and a study done in Arbanchi, Ethiopia (58). This may be because the first viral load measurement was done at the same time as the initiation of treatment (prospective study up to 6 months) at the prior study. This means the viral load might be high irrespective of the WHO stage. As this study is conducted after 6 months of treatment initiation, having a low WHO stage may linked with sharp decrease of viral load over 6 months (60).

Taking ART service outside the catchment area was associated with a high log viral load at six months post-treatment initiation. This may be due to missing their appointment date and treatment interruption, which in turn leads to adherence problems and treatment failure. Another possible explanation may be fear of disclosure to the public due to discrimination and stigmatization (60).

Poor adherence level is associated with high log viral load at six months post-treatment initiation. This study is supported by studies conducted in Arbaminch and Zewuditu Memorial Hospital, Ethiopia (27, 58, 59). This may be due to the inability to take medication properly; this affects the efficacy of HAART medication on viral load reduction and leads to resistance to HAART medication that may result in treatment failure.

Individual patient log viral load change before 10.82 months of follow-up deviates from the average population log viral load change is associated with time to death. This finding is supported by studies conducted in the United States, sub-Saharan Africa, and South Africa (32, 56, 61, 62). This may be due to differences in the viral load change between patients who survived and those who died (63, 64).

## Strengths and limitations of the study

This study uses robust analysis without constraining the natural follow of the data using random visit times. This is achieved using Bayesian analysis, incorporating a skewness parameter, modeling undetectable viral load with the Tobit mixed-effects model, and handling missing data through joint analysis. The study findings apply to other ART treatment sites in Ethiopia since all study centers have similar findings on viral load change, given the generalizability to similar populations (proximal population generalizability). These findings may also be relevant for other developing countries that follow the WHO guidelines.

The weakness of this study is its inability to include time interaction effects of baseline covariates on viral load change due to the slow execution of the software. Few viral load measurements per participant may affect the findings. The viral load measurements were not measured at the planned time points (viral load measurements at 6 months, 12 months, and every 12 months since treatment initiation), and many patients had only two and three viral load measurements within a 4-year follow-up period. In fact, a minimum of five viral load measurements were considered, and more for those who showed immunologic, clinical, and virologic failure.

## Implication of the study

The study may be used by policymakers for guideline revision or modification to incorporate the viral load change and factors associated with viral load rebound into the program to overcome resource-intensive viral load measurement as well as to prevent HIV transmission due to high viral load measures along with protective measures.

The study may be used by clinicians to assess the prognosis of their clients by observing the speed of viral load change, assess the clients in time to prevent transmission among couples and high-risk groups, identify treatment failure as early as possible, and highlight the factors associated with viral load change and survival. It will be used as a baseline for future studies on this area (viral load).

## Conclusion

Duration of treatment, WHO stages 3 and 4, taking Art service outside the catchment area, and poor adherence are associated with log viral load change. Log viral load decreases up to 11 months of follow-up, remaining low at the transition point and rebounding after 21 months. Individual patient log viral load change deviation from the mean log viral load change of the population is associated with time to death. The log viral load decrement after six months post-treatment initiation is smaller compared to the log viral load increment after 21 months since six months post-treatment initiation (27 months since treatment initiation).

## Data Availability

The datasets presented in this article are available upon reasonable request. Requests to access the datasets should be directed to eyobt525152@gmail.com.
