# Corrigendum: Viral load change and time to death among adult HIV/AIDS patients on ART after test-and-treat in Northwest Ethiopia: a retrospective multi-center follow-up study using Bayesian joint modeling

**DOI:** 10.3389/fpubh.2025.1621523

**Published:** 2025-05-14

**Authors:** Eyob Tilahun Abeje, Eskezyiaw Agedew, Bekalu Endalew, Gedefaw Diress Alen

**Affiliations:** ^1^Department of Epidemiology and Biostatistics, School of Public Health, College of Medicine and Health Sciences, Wollo University, Dessie, Ethiopia; ^2^Department of Public Health, College of Health Sciences, Debre Markos University, Debre Markos, Ethiopia

**Keywords:** viral load change, viral load pattern, time to death, HIV/AIDS, viral load rebound, survival analysis, Bayesian analysis

In the published article, there was an error in Figure 2 as published. The x-axis did not display ticks at consistent 6-month intervals (from 0 to 42 months). Additionally, the Figure 2 title “Viral load trajectories” was missed. The corrected Figure 2 and its caption appear below.

**Figure 2:**
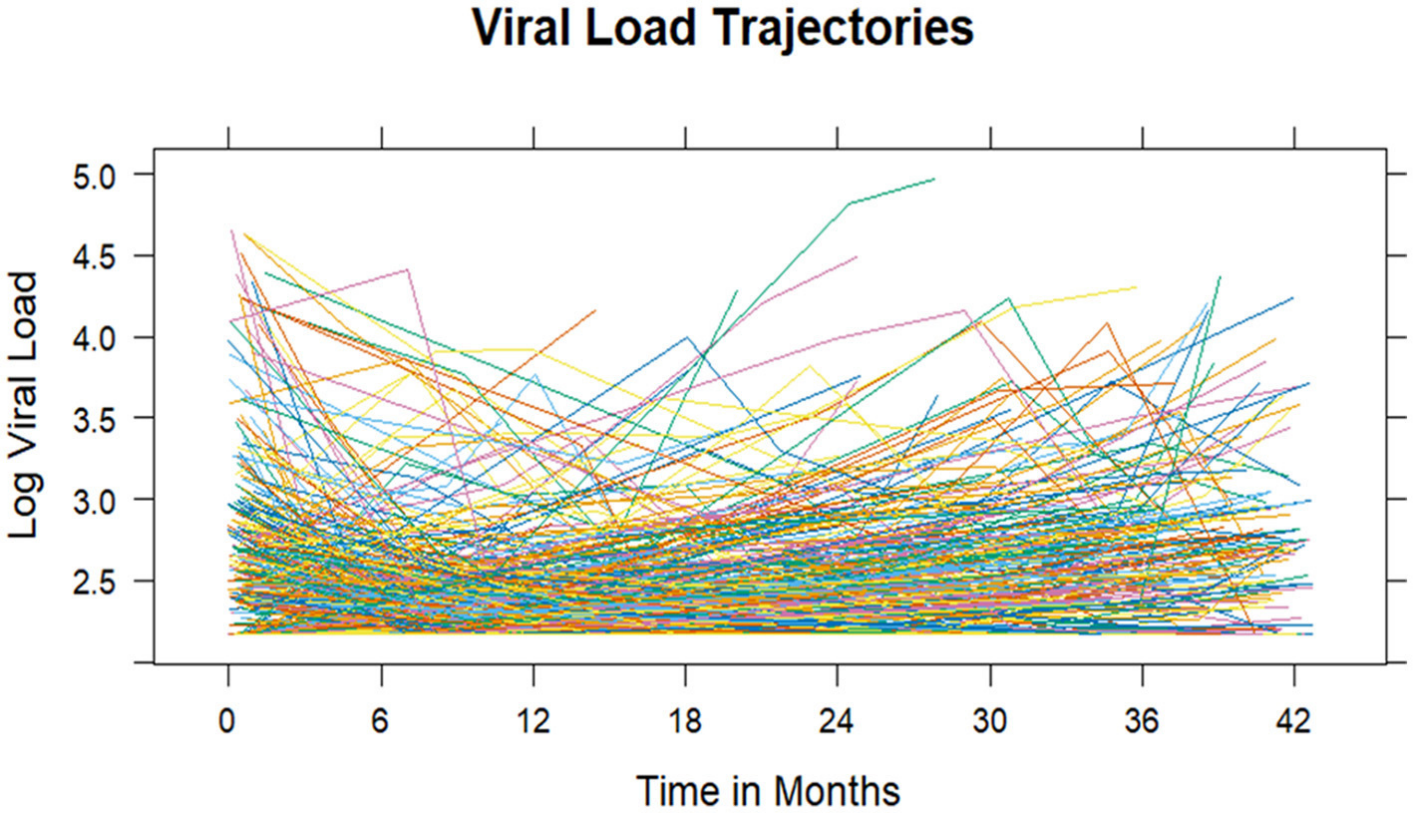
Viral load trajectory: individual profile plots.

The authors apologize for this error and state that this does not change the scientific conclusions of the article in any way. The original article has been updated.

